# Immunohistochemical profile of non-invasive follicular thyroid neoplasm with papillary-like nuclear features (NIFTP) versus other thyroid follicular lesions

**DOI:** 10.1186/s13000-025-01660-z

**Published:** 2025-05-26

**Authors:** Rehab Monir Samaka, Aiat Shaban Hemida, Hagar Alfouly, Mona A. Kora

**Affiliations:** Pathology Department, Faculty of Medicine, Minufiya University, Menoufia, Shebin El-kom, 332511 Egypt

**Keywords:** Diagnosis, Thyroid follicular lesions, NIFTP, RRM2, APLP2, CD56 and HBME-1

## Abstract

**Background:**

A follicular thyroid tumour called Non-invasive follicular thyroid neoplasm with papillary-like nuclear features (NIFTP) poses crossing-over morphologic characteristics with more thyroid follicular lesions whether benign or cancerous nodules. This study focuses on analysing the expression of CD56, HBME-1, RRM2 and APLP2 IHC markers in NIFTP versus other thyroid follicular lesions and their diagnostic validity was also evaluated.

**Methods:**

one hundred and nine thyroidectomy specimens including 31 NIFTP, 34 non-neoplastic, 34 papillary thyroid carcinoma (PTC) and 10 invasive encapsulated follicular variant papillary thyroid carcinoma (IEFVPTC) cases, were acquired between 2019 and 2022 from the Menoufia University’s Faculty of Medicine’s Pathology Department. Tissue microarray construction (TMA) blocks were prepared and CD56, HBME-1, RRM2 and APLP2 immunostaining were performed.

**Results:**

For CD56, 64.5% of NIFTP, 97.1% of the non-neoplastic group and 0% of both PTC and IEFVPTC were positive. For HBME-1, 61.3% of NIFTP, 0% of non-neoplastic, 100% of PTC and 100% of IEFVPTC were positive. For RRM2, all cases of NIFTP and the non-neoplastic group were negative, 88.2% of PTC and 100.0% of IEFVPTC were positive. For APLP2, 90.3% of NIFTP, 100% of the non-neoplastic group, 100% of PTC and 100% of IEFVPTC were positive. In differentiating NIFTP from non-neoplastic cases, the most sensitive marker was CD56 at H-score < 225 (sensitivity 95%) and the most specific was HBME-1 (specificity 100%). In various combinations, the panel of combined HBME-1 with either CD56 or APLP-2 improves their specificity (96.67% and 100% respectively) and the diagnostic accuracy (86.79 and 83.87, respectively) and therefore, combined HBME-1 and CD56 seems to be the most significant than using a single marker. In differentiation between NIFTP and PTC/IEFVPTC, the most sensitive marker was RRM2 (100% sensitivity for both groups) with the highest diagnostic accuracy (93.85% and 100%, respectively) and the most specific was CD56 (specificity 100% for both groups).

**Conclusions:**

Immunohistochemical markers such as CD56, HBME-1, RRM2, and APLP2 may aid in the diagnosis of NIFTP and its distinction from other follicular lesions.

## Introduction

One of the ten most prevalent malignancies in the United States is thyroid cancer. Over the previous three decades, thyroid cancer has become more common; 44,280 additional cases are predicted in 2024 with female predominance being three times more in females than males [1]. These increasing rates are claimed to be due to overdiagnosis of papillary thyroid carcinoma (PTC) [2]. Primary malignant thyroid neoplasms accounted for 74.7% of malignant endocrine tumours and 1.96% of all malignant neoplasms at the National Cancer Institute (NCI) in Egypt. Seventy per cent of all initial malignant thyroid tumours are PTC [3].

Among differentiated thyroid cancers (DTC), PTC is the most prevalent type [4, 5], and its follicular variation (FVPTC) has emerged as the most prevalent architectural pattern representing 57% and showing increasing rates in the last decades [5]. Since the non-invasive variant of encapsulated FVPTC (NI-EFVPTC) was being overtreated and demonstrated indolent behaviour, the name “Non-invasive follicular thyroid neoplasm with papillary-like nuclear features (NIFTP)” was proposed [6].

A full capsule with a distinct separation of the tumour from the surrounding thyroid, no capsule invasion, a mostly or solely follicular growth pattern, and nuclear characteristics of PTC are the histologic criteria for diagnosing NIFTP [7, 8]. Attention has turned to immunohistochemical (IHC) markers to distinguish between benign and malignant thyroid follicular lesions and different follicular neoplasms since benign and malignant thyroid follicular lesions often share morphologic and architectural similarities, making diagnosis challenging. Hector Battifora mesothelial-1 (HBME1), Fibronectin1 (FN1), Cytokeratin 19 (CK19), Cbp/p300-interacting trans-activator with Glu/Asp-rich carboxy-terminal domain1 (CITED1), Cluster of Differentiation (CD56), and Galectin3 (GAL3) are among the IHC markers that have been studied as possible diagnostic markers for PTC [9–14]. Among these CD56 and HBME-1 have gained popularity. Moreover, Amyloid precursor-like protein 2 (APLP2), Ribonucleotide reductase M2 polypeptide (RRM2) and Protein Regulator of cytokinesis 1 (PRC1) are new indicators studied to differentiate thyroid follicular lesions [15].

The objective of this research is to examine the expression of CD56, HBME-1, APLP2, and RRM2 IHC markers in NIFTP and the efficacy of these markers in distinguishing between benign and malignant follicular lesions.

## Methods

### Sample size estimation

The estimated sample size is 25 cases per group, considering NIFTP as the case and non-neoplastic, PTC, and IEFVPTC cases as a control group. The sample size was obtained from the Public Health and Community Medicine Department at our University.

### Study participants

This research comprised 109 thyroidectomy specimens in a retrospective case-control design. The studied cases were categorized into a case group (31 NIFTP) and a control group that included 34 non-neoplastic, 34 PTC cases and 10 invasive encapsulated follicular variants of PTC (IEFVPTC).

From 2019 to 2022, tissue blocks that had been paraffin-embedded and formalin-fixed were extracted from the archived material that was acquired from the Pathology Department of Menoufia University’s Faculty of Medicine. They were chosen because paraffin-embedded blocks were available for serial cutting and analysis. Information gathered from the patients’ medical records comprised: age, sex and specimen type.

### Histopathological evaluation

Histopathological evaluation was performed by an expert pathologist to characterize tumours in each category. The fifth World Health Organization (WHO) classification system’s diagnostic criteria served as the basis for the diagnosis [8]. NIFTP case diagnosis is based on the revised inclusion and exclusion criteria designated in 2018 and evaluated with the publishment of the 2022 WHO Classification of Thyroid Neoplasms [7, 8].

### Tissue microarray (TMA) blocks and IHC staining

The best representative possible area was obtained from each block and using a manual tissue microarray (Breecher instrument Manual Microarray, Wisconsin, USA), duplicate-core TMA blocks were processed.

The automated LINK 48 immunostainer (Dako, Agilent Technologies Inc., Santa Clara, USA) was used to stain the sections after they were cut at 3 μm. Citrate buffer was employed to recover heat. Primary diluted antibodies were used to automatically dye the slides including, CD56 (Catalog No. ab22036, Abcam biotechnology Co., 1:100 dilution (, HBME-1 (Catalog No. ab2383, Abcam biotechnology Co., 1:50 dilution(, RRM2 (Catalog No. YPA2409, Chongqing biospes Vo., Ltd, 1:100 dilution( and APLP2 (Catalog No. YPA2408, Chongqing biospes Vo., Ltd, 1:150 dilution). Positive control for CD56 is pancreas, HBME1 is mesothelioma, RRM2 is invasive breast cancer and APLP2 is normal colonic mucosa. Non-immune serum was used in place of the main antibodies to provide negative controls.

Positive expression for both CD56 and HBME-1 immunostaining should be considered if > 10% of cells showed positive brownish membranous staining with or without cytoplasmic staining [16–18]. For APLP2 and RRM2, when cells exhibit nuclear brownish staining, whether or not cytoplasmic staining is present, positive expression should be taken into account [15]. The following formula was used to determine each marker’s H-score: One per cent of mildly stained cells plus two per cent of moderately stained cells plus three per cent of strongly stained cells equals the H-score [19].

### Statistical data analysis

IBM SPSS software package version 20 (Armonk, NY: IBM Corp.) was used to analyze the data that was input into the computer. Numbers and percentages were used to describe the qualitative data. The data distribution’s normality was confirmed using the Shapiro-Wilk test. Range (minimum and maximum), mean, standard deviation (SD), median, and interquartile range (IQR) were used to characterize quantitative data. Statistical significance was defined as a p-value < 0.05.

Kruskal Wallis test, the Mann-Whitney test, the Chi-square test, and Fisher’s Exact or Monte Carlo correction were among the employed tests. Plotting sensitivity (TP) on the Y axis against 1-specificity (FP) on the X axis at various cut-off settings produced the receiver operating characteristic curve (ROC). The test’s diagnostic performance is shown by the area under the ROC curve. A result of over 50% is considered acceptable, whereas the best performance for the test is almost 100%. It is also possible to compare the performance of two tests using the ROC curve. Traditional diagnostic test formulas were used to determine sensitivity, specificity, positive predictive value (PPV), negative predictive value (NPV), and diagnostic accuracy.

## Results

### Clinicopathological data of studied groups

The demographic information for the patients in this investigation is gathered in Table 1 (*n* = 109).

**Table 1:** Summary of clinicopathological features of studied groups

Parameters	NIFTP (*n* = 31)	Non-neoplastic (*n* = 34; MNG = 19, FA = 6, HT = 9)	Conventional PTC(*N* = 34)	IEFVPTC (*N* = 10)
**Gender**				
M/F ratio	1:30	3:31	4:13	1:04
**Age**				
Min. – Max.	17–69	20–85	17–75	26–51
Mean ± SD.	35.65 ± 14.34	44.59 ± 14.17	44.1 ± 16.44	39.2 ± 9.8
Median (IQR)	32 (23.5–44)	43(35–53)	42.5(29–54)	43 (31–45)
**Type of specimen**				
Hemi thyroidectomy	10 (32.3%)	8(23.5%)	3(8.8%)	4 (40%)
Total thyroidectomy	21 (67.7%)	26(76.5%)	31(91.2%)	6 (60%)
**Laterality of the lesion**				
Unilateral	27 (87.1%)	-	20(58.8%)	9 (90%)
Bilateral	4 (12.9%)		14(41.2%)	1 (10%)
**Size (The largest dimension in cm)**				
Min. – Max.	0.2–7	-	1–11	1.0–3.5
Mean ± SD.	3.3 ± 1.89		3.33 ± 2.27	2.4 ± 0.9
Median (IQR)	3.5 (2–5)		3(1.5–4)	2.5 (4–4)
**Focality of the lesion**				
Solitary	25(80.6%)	-	18(52.9%)	9 (90%)
Multifocal	6 (19.4%)		16(47.1%)	1 (10%)
**Goss pattern**				
Well-demarcated	3 (9.7%)	-		0 (0%)
Capsulated	28 (90.3%)			10 (100%)
**Type of capsule (No = 28)**		-		
Partial capsule	14 (50%)			2 (20%)
Complete capsule	14 (50%)			8 (80%)
**Capsular invasion (No = 28)**		-		
Present	0 (0%)			10 (100%)
Absent	28 (100)			0 (0%)
**Psammoma bodies**		-		
Present	0 (0%)		12 (35.3%)	2 (20%)
Absent	31 (100%)		22 (64.7%)	8 (80%)
**Thyroid capsular invasion**				
Present	0 (0%)	-	2 (5.9%)	0 (0%)
Absent	31 (100%)		32 (94.1%)	10 (100%)
**Lymphovascular invasion**		-		
Present	0 (0%)		3 (8.8%)	0 (0%)
Absent	31 (100%)		31 (91.2%)	10 (100%)
**Tumor necrosis**				
Present	0 (0%)	-	0 (0%)	0 (0%)
Absent	31 (100%)		34 (100%)	10 (100%)
**Number of mitoses per 2 mm** ^**2**^				
Min. – Max.	0–2	-	0–3	0–3
Mean ± SD.	0.77 ± 0.62		1.38 ± 0.7	1.38 ± 0.7
Median (IQR)	1(0–1)		1(1–2)	1(1–2)
**Pathological TNM stage**				
PT1		-	16 (47.1%)	4 (40%)
PT2	-		13 (38.2%)	6 (60%)
PT3			5 (14.7%)	0
N0			24 (70.6%)	10(100%)
N1	-	-	10(29.4%)	0
M0			34(100%)	10(100%)
**AJCC prognostic stage grouping**	-	-		
Stage I			28 (82.4%)	10(100%)
Stage II			6 (17.6%)	0

IQR: Inter quartile range SD: Standard deviation U: Mann Whitney test c^2^: Chi-square test MC: Monte Carlo

p: p-value for comparing between the three studied groups

p_1_: p-value for comparing between NIFTP and Non-neoplastic

p_2_: p-value for comparing between NIFTP and PTC

p_3_: p-value for comparing between NIFTP and IEFVPTC

p_4_: p-value for comparing between Non-neoplastic and PTC

P5: p-value for comparing between Non-neoplastic and IEFVPTC

p_6_: p-value for comparing between PTC and IEFVPTC

*: Statistically significant at *p* ≤ 0.05

### Comparison between studied groups according to CD56, HBME-1, RRM2 and APLP2 expression

**Fig. 1:**
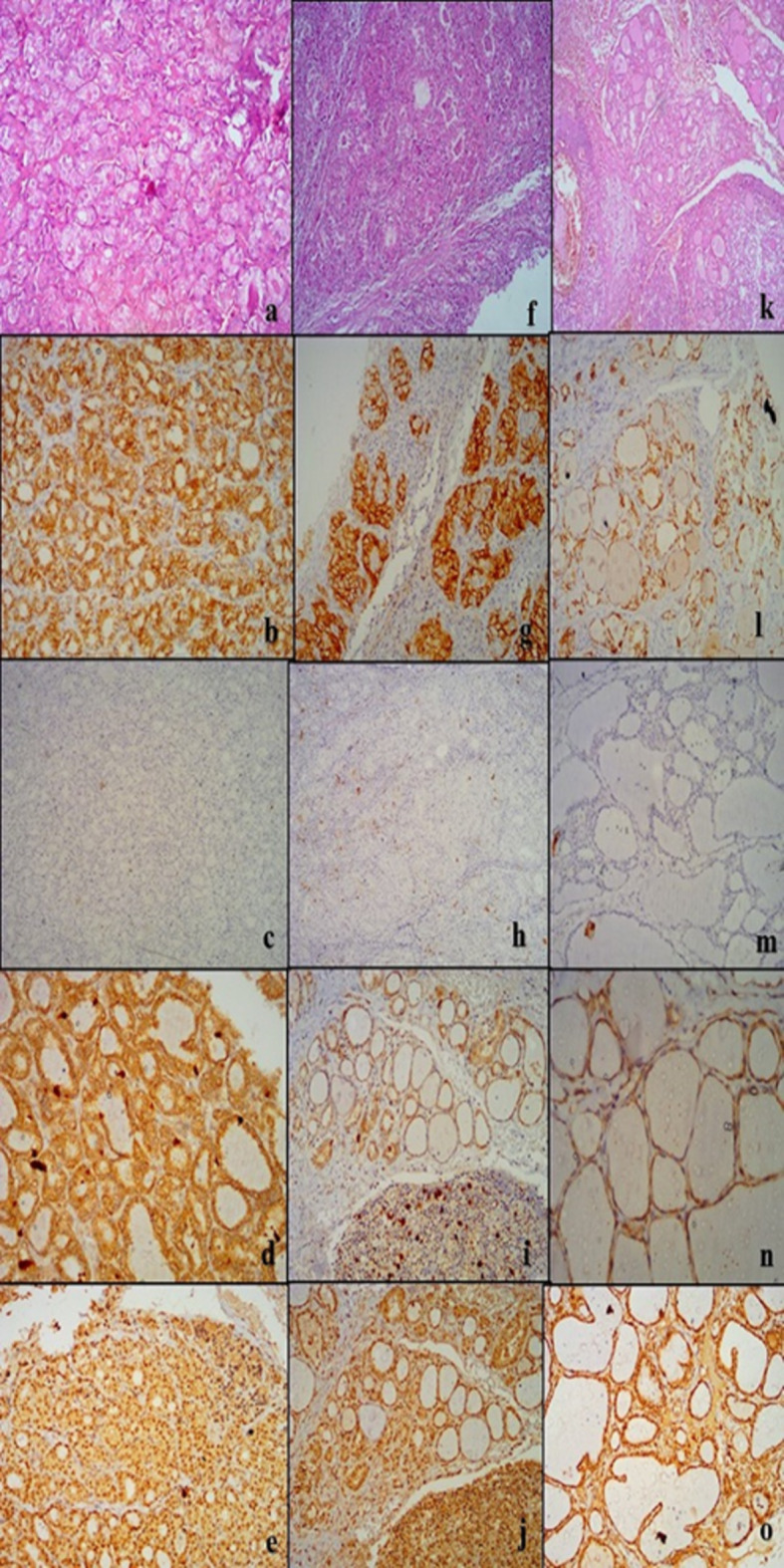
Microscopic results of the four IHC markers in the non-neoplastic group. Hematoxylin-eosin (HE) stain for follicular adenoma (FA) (**a**), Hashimoto thyroiditis (HT) (**f**) and Multinodular goitre (MNG) (**k**). CD56 showed diffuse membranous staining in FA (**b**), HT (**g**) and MNG (**l**). HBME-1 was negative in FA (**c**), HT (**h**) and MNG (**m**). RRM2 exhibited negative nuclear staining with non-specific cytoplasmic staining in FA (d), HT (**i**) and MNG (**n**). APLP2 with positive atomic staining and nonspecific cytoplasmic staining in FA (**e**), HT (**j**) and MNG (**o**). Magnification was x100 for all

**Fig. 2:**
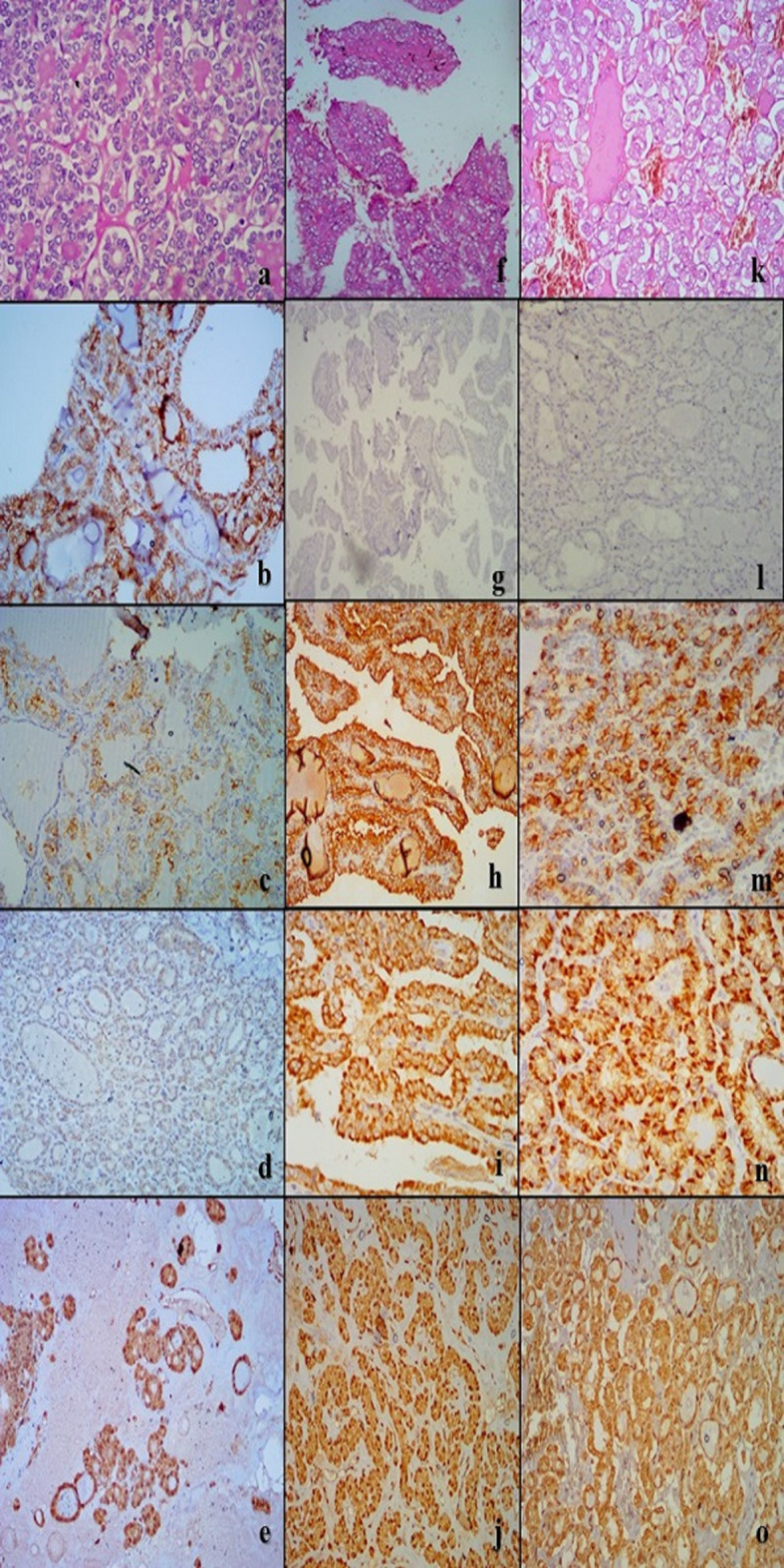
Microscopic images of the four IHC markers in NIFTP, PTC and IEFVPTC. Hematoxylin–eosin (HE) stain for NIFTP (**a**), PTC (**f**) and IEFVPTC (**k**). CD56 showed diffuse membranous staining in NIFTP (**b**), and negative staining in PTC (**g**) and IEFVPTC (**l**). HBME-1 showed focal positive membranous staining in NIFTP (**c**), diffuse staining in PTC (**h**) and IEFVPTC (**m**). RRM2 exhibited negative nuclear staining with non-specific cytoplasmic staining in NIFTP (**d**), positive nuclear staining in PTC (**i**) and IEFVPTC (**n**). APLP2 with positive nuclear staining and nonspecific cytoplasmic staining in NIFTP (**e**), negative nuclear staining in PTC (**j**) and IEFVPTC (**o**). Magnification was x100 for all

**Fig. 3:**
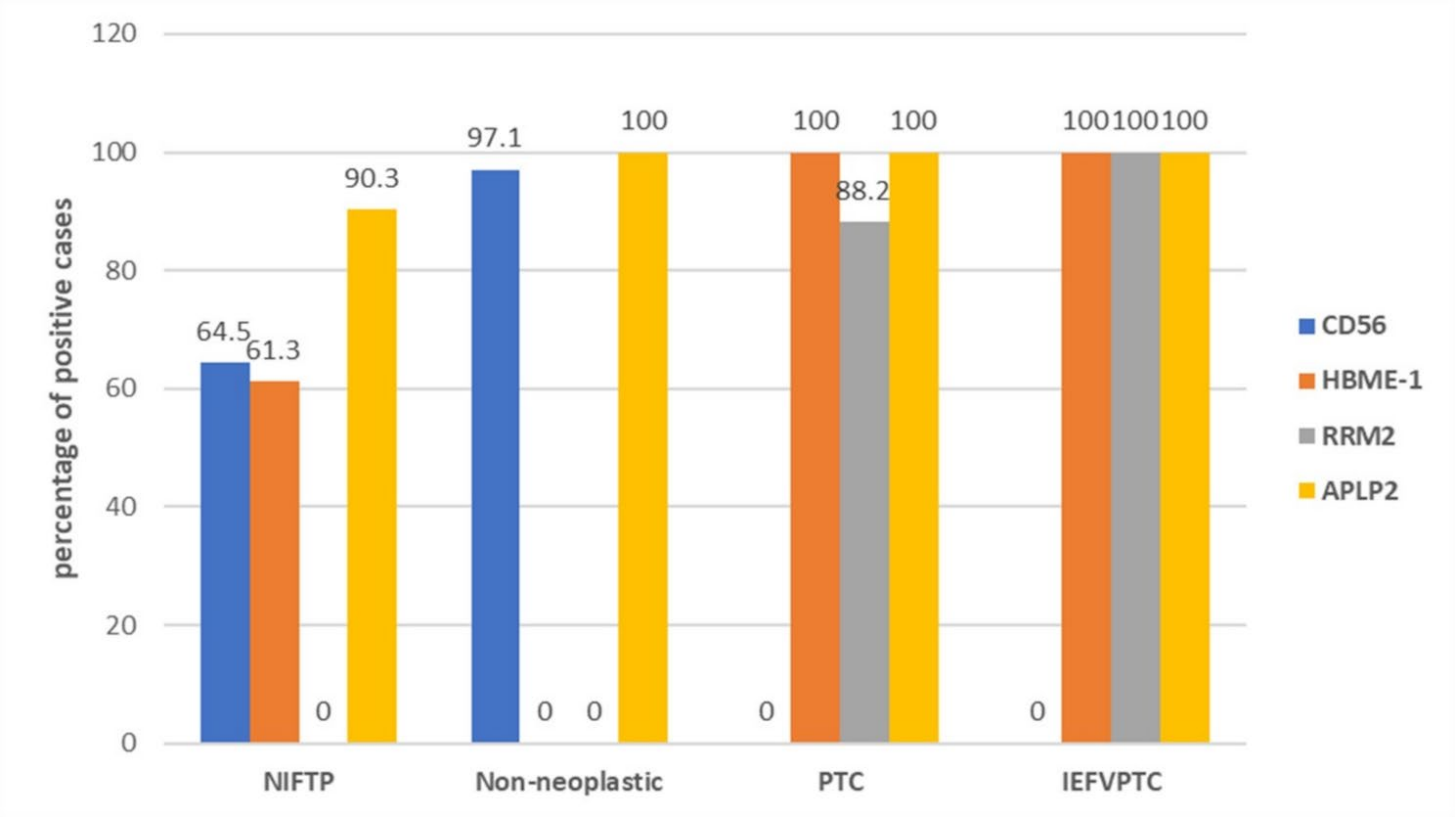
Cross-classification between all studied groups and status of the four studied markers

Table 2 outlines the results for IHC expression of CD56, HBME-1, RRM2, and APLP2 in the various diagnostic classifications. Regarding CD56, 64.5% of NIFTP cases were positive, 97.1% of non-neoplastic cases were positive with strong intensity in 84.8% of non-neoplastic positive cases compared to 40% of NIFTP, while all PTC (conventional and IEFVPTC) cases were negative. Regarding HBME-1, 61.3% of NIFTP, all PTC and IEFVPTC were positive with strong staining in (97.1%) of IEFVPTC and all PTC compared to 68.4% of NIFTP cases, while all non-neoplastic group was negative. Considering RRM2, all NIFTP and non-neoplastic cases were negative while all IEFVPTC and 88.2% of PTC were positive. For APLP2, its positivity showed no significance in differentiating between studied groups. Figures 1, 2 and 3.

**Table 2:** Comparison between studied groups according to the expression of CD56, HBME-1, RRM2 and APLP2

	NIFTP (No = 31)	Non-neoplastic (No = 34)	PTC (No = 34)	IEFVPTC (No = 10)	Test of Sig.	*p*
	No. (%)	No. (%)	No. (%)	No. (%)		
**CD56**						
**Status**						
Positive	20 (64.5%)	33 (97.1%)	0 (0%)	0 (0%)	χ^2^= 76.706^*^	< 0.001^*^
Negative	11 (35.5%)	1 (2.9%)	34 (100%)	10 (100%)		
**Sig.bet.Groups**	P_1_ = 0.001^*^, P_2_ < 0.001^*^, ^FEP^_3_<0.001^*^,P_4_ < 0.001^*^,^FE^P_5_<0.001^*^					
**Dominant intensity**						
Mild	9 (45%)	1 (3%)	–	–	χ^2^= 15.075^*^	^MC^p < 0.001^*^
Moderate	3 (15%)	4 (12.1%)	–	–		
Strong	8 (40%)	28 (84.8%)	–	–		
**H score**						
Min. – Max.	25–235	55–285	–	–	U= 121.0^*^	< 0.001^*^
Mean ± SD.	124.45 ± 77.36	212.7 ± 60.64	–	–		
Median (IQR)	145 (47 − 200)	230 (170 − 260)	–	–		
**HBME-1**						
**Status**						< 0.001^*^
Positive	**19 (61.3%)**	**0 (0%)**	**34 (100%)**	**10 (100%)**	χ^2^= 78.847^*^	
Negative	12 (38.7%)	34 (100.0%)	0 (0%)	0 (0%)		
**Sig. bet.Groups**	P_1_ < 0.001^*^,P_2_ < 0.001^*^,^FE^P_3_=0.021^*^,P_4_ < 0.001^*^,^FE^P_5_<0.001^*^_,_p_6_=–					
**Dominant intensity**						
Mild	1 (5.3%)	–	0 (0%)	0 (0%)	χ^2^= 9.714^*^	^MC^p= 0.014^*^
Moderate	5 (26.3%)	–	1 (2.9%)	0 (0%)		
Strong	13 (68.4%)	–	33 (97.1%)	10 (100%)		
**Sig. bet.Groups**	p_1_–, ^MC^p_2_=0.007^*^, ^MC^p_3_=0.179, p_4_–, p_5_=–,p_6_ = 1.000					
**H score**						
Min. – Max.	25–230	–	45–285	45–265	H= 12.267^*^	0.002^*^
Mean ± SD.	137.6 ± 62.28	–	204.3 ± 648	200.5 ± 67.47		
**RRM2**						
**Status**					χ^2^= 93.807^*^	< 0.001^*^
Positive	**0 (0%)**	**0 (0%)**	**30 (88.2%)**	**10 (100.0%)**		
Negative	31 (100.0%)	34 (100.0%)	4 (11.8%)	0 (0%)		
**Sig. bet.Groups**	P1:NA, P_2_ < 0.001^*^_,_^FE^P_3_<0.001^*^,P_4_ = < 0.001^*^,^FE^P_5_<0.001^*^,^FE^P_6_=0.559					
**Dominant intensity**					χ^2^= 0.048	^FE^p= 1.000
Mild	–	–	0 (0%)	0 (0%)		
Moderate	–	–	7 (23.3%)	2 (20.0%)		
Strong	–	–	23 (76.7%)	8 (80.0%)		
**H score**					U= 144.0	0.866
Min. – Max.	–	–	35–265	85–210		
Mean ± SD.	–	–	136.83 ± 60.09	142 ± 49.73		
Median (IQR)	–	–	137.5(100–180)	135(100–180)		
**APLP2**						
**Status**					χ2= 4.938*	
**Positive**	28 (90.3%)	34 (100%)	34 (100%)	10 (100%)		MCp= 0.044*
**Negative**	3 (9.7%)	0 (0%)	0 (0%)	0 (0%)		
**Sig. bet.Groups**	FEP1 = 0.103, FEP2 = 0.103, FEP3 = 0.564,p4=–,p4 = p5=–,p6=–					
**Dominant intensity**					χ2= 64.202*	MCp < 0.001*
**Mild**	7 (25%)	0 (0%)	25 (73.5%)	9 (90%)		
**Moderate**	11 (39.3%)	9 (26.5%)	6 (17.6%)	0 (0%)		
**Strong**	10 (35.7%)	25 (73.5%)	3 (8.8%)	1 (10%)		
**Sig. bet.Groups**	MCp1 < 0.001*,p2 < 0.001*,p3 < 0.001*,p4 < 0.001*,MCp5 < 0.001*,MCp6 = 0.455					
**H score**						< 0.001*
**Min. – Max.**	3–280	40–280	30–250	50–190	H= 36.620*	
**Mean ± SD.**	136.1 ± 87.83	209.5 ± 59.94	101.9 ± 572	73.50 ± 44.22		
**Median (IQR)**	147(55 − 202.5)	225 (170–265)	80 (60 − 165)	50(50–75)		
**Sig. bet.Groups**	p1 < 0.001*,p2 = 0.123,p3 = 0.045*,p4 < 0.001*,p5 < 0.001*,p6 = 0.338					

### The four indicators’ diagnostic accuracy both separately and together in discriminating NIFTP from control groups (ROC curve analysis results)

**Fig. 4:**
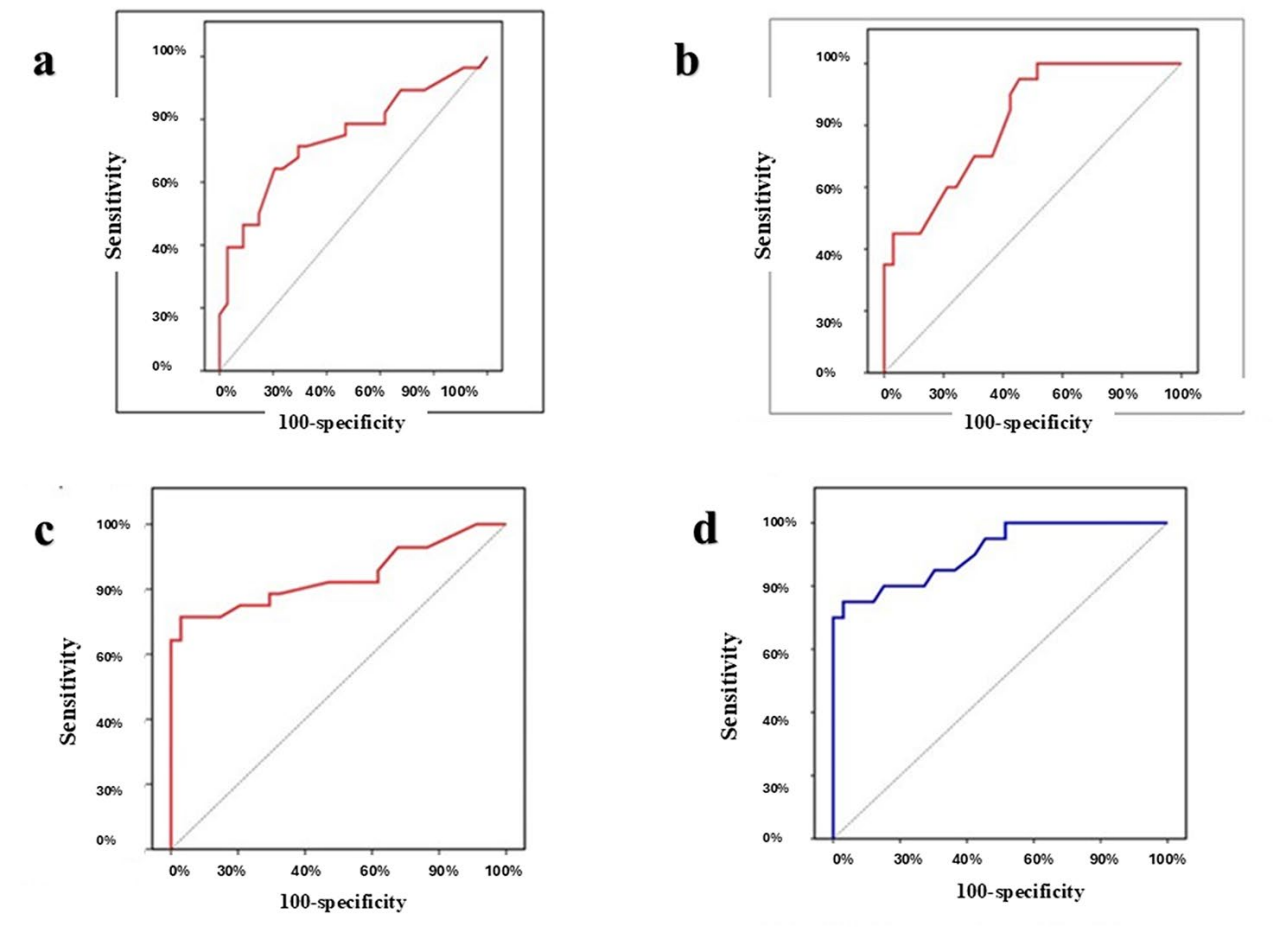
ROC curves of discrimination between NIFTP and non-neoplastic cases. (**a**) ROC curve for H- score of APLP2 to discriminate NIFTP from Non neoplastic. (**b**) ROC curve for H-score of CD56 to discriminate NIFTP from Non neoplastic. (**c**) ROC curve for combined APLP2 H-score + HBME-1 status to discriminate NIFTP from Non neoplastic. (**d**) ROC curve for combined CD56 H-score + HBME-1 status to discriminated NIFTP from non neoplastic

To distinguish NIFTP from the non-neoplastic group, the diagnostic accuracy of the four markers both separately and in combination is shown in Table 3; Fig. 4.

**Table 3:** Diagnostic accuracy of the four markers alone and in combination to discriminate NIFTP from non-neoplastic

IHC Markers	AUC	*P*	95% C.I	Sensitivity	Specificity	PPV	NPV	Accuracy
**APLP2 H score at cut off (< 250)**	0.743	< 0.001^*^	0.615–0.871	89.29	32.35	52.1	78.6	58.06
**CD56 H-score at cut-off ≤ 225**	0.817	< 0.001*	0.705–0.928	95	54.55	55.9	94.7	69.81
**HBME-1 status**		< 0.001*		61.29	100	100	73.91	81.54
**Combined H score of APLP2 (cut off ≤ 250) + Status HBME-1**	0.839	< 0.001*	0.729–0.948	64.29	100	100.0	77.27	83.87
**Combined H score of CD56 (cut off ≤ 225) + Status HBME-1**	0.910	< 0.001*	0.828–0.992	70	96.67	93.33	84.21	86.79

AUC: Area Under Curve p-value: Probability value CI: Confidence Intervals

NPV: Negative predictive value PPV: Positive predictive value *: Statistically significant at *p* ≤ 0.05

**Fig. 5:**
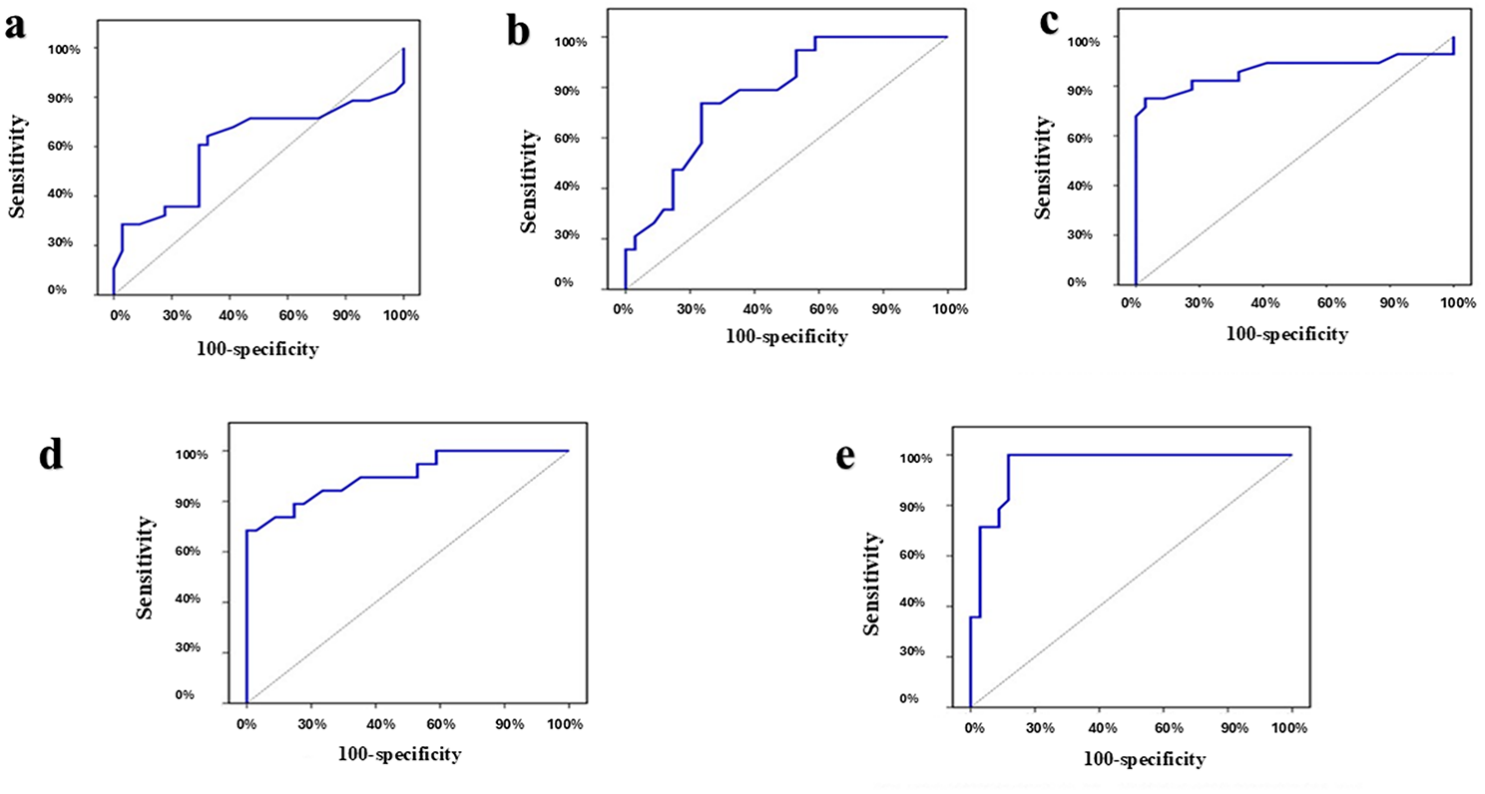
ROC curves to distinguish between PTC and NIFTP. (**a**) ROC curve for H-score of APLP2. (**b**) ROC curve for H-score of HBME-1. (**c**) ROC curve for combined H-score APLP2 + CD56 status. (**d**) ROC curve for combined H-score HBME-1 + CD56 status. (**e**) ROC curve for combined H-score APLP2 + RRM2 status

The diagnostic accuracy of the four indicators, both separately and in combination, to distinguish NIFTP from PTC is shown in Table 4; Fig. 5.

**Table 4:** Diagnostic accuracy of the four markers alone and in combination to discriminate NIFTP from PTC

	AUC	*P*	95% C.I	Sensitivity	Specificity	PPV	NPV	Accuracy
**APLP2 H-score cut off > 45**	0.605	0.157	0.453–0.757	78.57	11.76	42.3	40.0	41.93
**HBME-1 H-score cut off ≤ 220**	0.778	0.001*	0.654–0.902	94.74	47.06	50.0	94.1	64.15
**CD56 status**		< 0.001*		64.52	100.0	100.0	75.56	83.08
**RRM2 status**				100	88.24	88.57	100.0	93.85
**Combined H score APLP2 cut off > 45 +CD56 status**	0.863	< 0.001*	0.752–0.974	67.86	100.0	100.0	79.07	85.48
**Combined H score HBME-1 cut off ≤ 220 + CD56 status**	0.902	< 0.001*	0.813–0.992	68.42	100.0	100.0	85.0	88.68
**Combined H score APLP2 cut off > 45 + Status RRM2**	0.959	< 0.001*	0.911–1.0	100.0	88.24	87.50	100.0	93.55

AUC: Area Under Curve p-value: Probability value CI: Confidence Intervals

NPV: Negative predictive value PPV: Positive predictive value *: Statistically significant at *p* ≤ 0.05

**Fig. 6:**
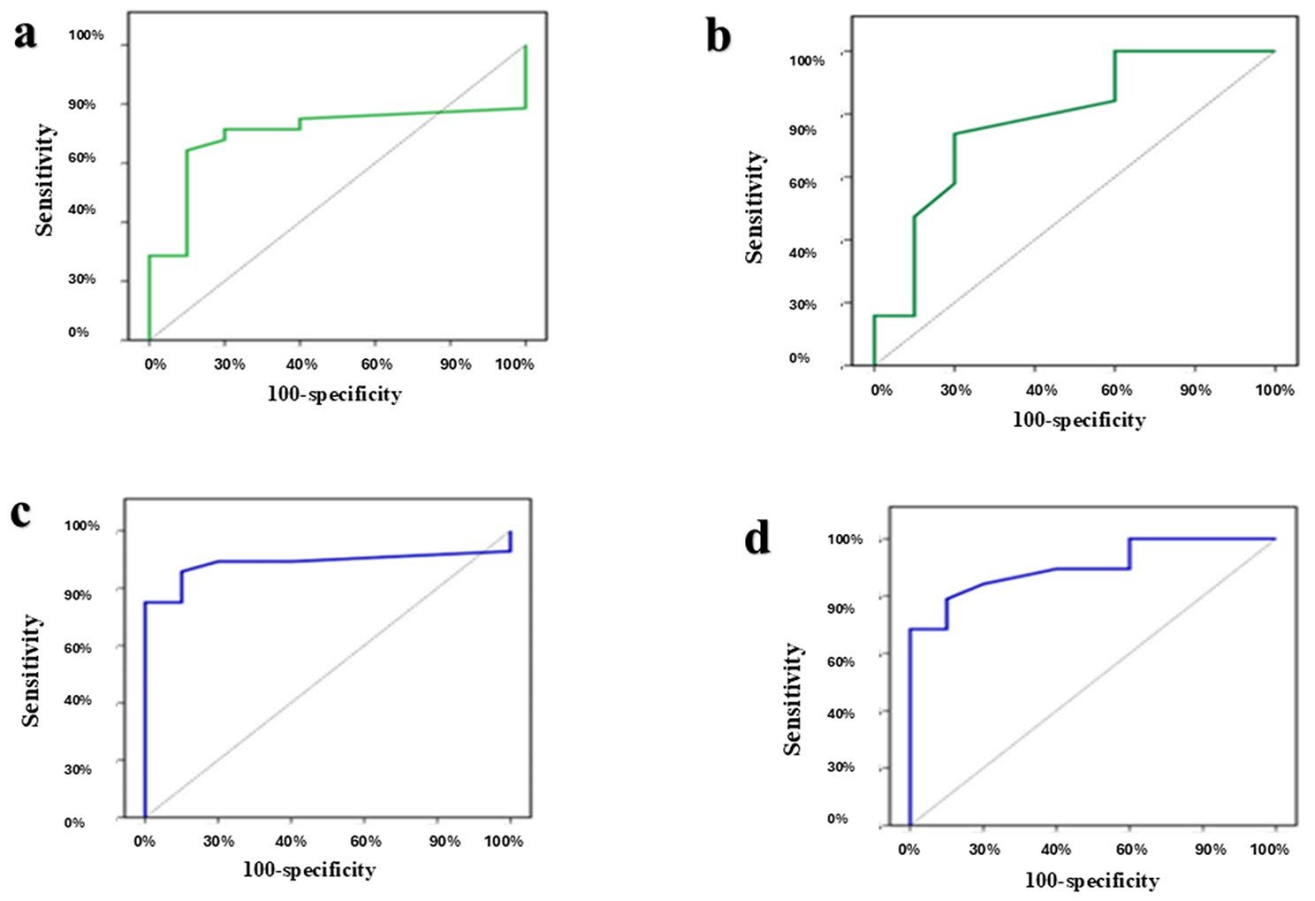
ROC curves to differentiate IEFVPTC from NIFTP. (**a**) ROC curve for H-score of APLP2. (**b**) ROC curve for H-score of HBME-1. (**c**) ROC curve for combined H-score APLP2 + CD56 status. (**d**) ROC curve for combined H-score HBME-1 + CD56 status

The diagnostic precision of the four markers, both separately and in combination, to distinguish NIFTP from IEFVPTC is shown in Table 5; Fig. 6.

**Table 5:** Diagnostic accuracy of the four markers alone and in combination to discriminate NIFTP from IEFVPTC

H score	AUC	*P*	95% C.I	Sensitivity	Specificity	PPV	NPV	Accuracy
APLP2 H score cut off > 50	0.698	0.066	0.527–0.869	75.0	60.0	84.0	46.2	71.05
HBME-1 H score cut off ≤ 220	0.784	0.013*	0.599–0.970	94.74	40.0	75.0	80.0	75.86
CD56 status				64.52	100	100	47.62	73.17
Status RRM2				100.0	100	100	100.0	100.0
Combined APLP2 H score cut off > 50 + CD56 status	0.808	< 0.001*	0.778–0.997	85.71	90	96	69.23	86.84
Combined HBME-1 H score cut off ≤ 220 + CD56 status	0.903	< 0.001*	0.794–1.0	78.95	90	93.75	69.23	82.76

AUC: Area Under Curve p-value: Probability value CI: Confidence Intervals

NPV: Negative predictive value PPV: Positive predictive value *: Statistically significant at *p* ≤ 0.05

## Discussion

Follicular patterned thyroid lesions including both benign lesions as multinodular goitre and follicular adenoma and malignant lesions as PTC, follicular variant of PTC and follicular carcinoma all have overlapping histologic features with NIFTP.While a diagnosis of PTC is typically made morphologically, using distinctive nuclear characteristics and IHC is seldom required to confirm the diagnosis [20, 21]. However, the nuclear features in NIFTP are usually subtle and focal so it can still be confused with encapsulated or well-demarcated classic PTC cases with predominant follicular patterns. FVPTC can be encapsulated (IEFVPTC) or infiltrative and widely differ in their morphology, exhibiting diffuse growth patterns and micro to macrofollicular structures. This can occasionally cause diagnostic confusion with NIFTP and other follicular neoplasms. As for follicular adenoma and follicular carcinoma, although nuclear features are absent in these lesions it can be confused with NIFTP because NIFTP nuclear features are usually subtle, focal and more in the periphery of the lesion. Also, molecular studies showed RAS mutations expressed heavily in follicular patterned thyroid tumours such as follicular adenoma, follicular carcinoma, NIFTP as well as FVPTC, so the NIFTP molecular profile is closer matched to these tumours. A similar molecular signature of NIFTP to IEFVPTC suggests that NIFTP may represent the “benign” precursor lesion of the IEFVPTC [22, 23]. So, the focus was shifted to the use of IHC indicators to differentiate between benign and cancerous lesions and distinguish the various follicular neoplasms. Our study evaluated the expression and potential diagnostic relevance of CD56, HBME1, RRM2, and APLP2 IHC markers in NIFTP in comparison to other benign and cancerous follicular lesions.

The membrane glycoprotein CD56 plays a key part in cell-cell adhesion. In individuals with certain malignant tumours such as PTC, pancreatic neuroendocrine neoplasm, small cell lung cancer and Merkel cell carcinoma, loss of CD56 expression has been linked to malignant transformation, tumour growth and a dismal prognosis [24–27]. Moreover, thyroid follicular epithelial cells exhibited strong CD56 membranous expression which decreased in malignant transformation [27]. So, CD56 was proved by many literatures to be a diagnostic marker with high specificity and sensitivity for benign thyroid lesions against malignant ones [14, 16, 18].

This study showed that all cases of PTC and IEFVPTC lacked positivity for CD56. Nearly all non-neoplastic cases showed diffuse strong membranous positivity with only one negative case. These results are similar to those reported in many previous literature [14, 16–18, 27–29]. Unlike our research, Etem et al. found that CD56 has no role in differentiating PTC from benign follicular lesions [30]. Regarding the NIFTP group, this thesis found that 64.5% of cases showed moderate to strong positive CD56 membranous staining. The intensity of CD56 expression showed a significant difference between NIFTP and non-neoplastic cases; whereas the majority of non-neoplastic cases were of strong intensity while 45% of NIFTP cases were mild. Moreover, the H-score showed a significant difference between NIFTP and the non-neoplastic group being of higher values in the non-neoplastic group. These findings are comparable to those of Chuang et al. [31] and Cho H et al. [32] where 60% and 82.4% of NIFTP cases were CD56 positive, respectively. However, one study done by Tastekin E et al., [33] showed that CD56 is positive in only 15% of NIFTP cases. CD56 was further investigated in our literature as a diagnostic marker differentiating NIFTP from non-neoplastic and malignant thyroid lesions. Given differentiating NIFTP from non-neoplastic cases, the best sensitivity of this marker for NIFTP was obtained at H-score < 225 which was 95% with a specificity of 54.5%. This specificity can be improved when CD56 is combined with HBME-1 to be 96.67% but at the expense of the sensitivity which was lowered to 70%. Given the difference between NIFTP and PTC/IEFVPTC, CD56 showed high specificity (100%) for NIFTP and less sensitivity (64.52%). The sensitivity can be slightly improved when CD56 is combined with HBME-1 or APLP2.

HBME-1 is considered a very valuable marker in differentiating malignant from benign lesions being strongly expressed in malignant follicular-derived thyroid neoplasm [18, 34, 35]. This was confirmed in our study as all PTC and IEFVPTC showed diffuse strong positive membranous staining with apical and lateral accentuation in many cases while all non-neoplastic cases were negative. According to Bachloc et al.‘s estimation, HBME-1’s sensitivity was 78.8% for thyroid cancer, 65.2% for follicular carcinomas, 87.3% for PTC and with a specificity of 82.1% [35]. Moreover, Sadiq et al. estimated HBME-1 sensitivity and specificity for PTC that was found to be 89%, 62% and 89%, 55% for FVPTC, respectively [36].

The majority of NIFTP cases in our literature (61.3%) showed strong to moderate membranous HBME-1 staining. Also cho et al., Sadiq et al. and Chuang et al. found similar results in which 64.4%, 77.8% and 56.2% of NIFTP were positive for HBME-1 [31, 32, 36]. In contrast to our findings, Tastekin et al. [33] found that HBME-1 is positive in only 13% of NIFTP cases.

Given the differentiating NIFTP from non-neoplastic lesions, the specificity of HBME-1 for NIFTP is 100% and the sensitivity is 61.29%. This sensitivity can be slightly improved when HBME-1 is combined with APLP2 and CD56. Regarding differentiating NIFTP from PTC/IEFVPTC, the best sensitivity of the marker to NIFTP (94.74%) is obtained at H-score cut-off ≤ 220 with specificity of 47.06% for PTC and 40% for IEFVPTC. According to these results, HBME-1 is a vulnerable marker in differentiating NIFTP from non-neoplastic cases with high specificity to NIFTP.At high H-score values, it can differentiate between NIFTP and malignant lesions (PTC/ IEFVPTC) with high sensitivity.

RRM2 is one of the two subunits of ribonucleotide reductase which catalyze nucleotides to deoxyribonucleotide triphosphate and so, important in DNA synthesis [37]. In thyroid, RRM2 nuclear overexpression was seen in PTC and anaplastic carcinoma [15, 38]. All non-neoplastic cases in our study and NIFTP cases were negative for RRM2; so, no role in differentiating NIFTP from non-neoplastic cases. PTC and IEFVPTC showed 88.2% and 100% moderate to strong nuclear RRM2 positivity, respectively. Agreeing with our results, Fang et al. found 44 out of 60 cases of thyroid cancer positive for RRM2 and none of the non-neoplastic cases were positive. Also, Castelblanco et al. found that RRM2 showed a significant difference between follicular adenoma and FVPTC [15, 38].

Our study is the first to examine RRM2 expression in NIFTP, as far as we are aware. The sensitivity and specificity of RRM2 to NIFTP against IEFVPTC were both 100%, also the sensitivity and specificity of RRM2 to NIFTP against PTC were 100% and 88.24%, respectively. Accordingly, RRM2 is considered a potential marker in differentiating malignant lesions (PTC/ EFVPTC) from either NIFTP or non-neoplastic lesions with high sensitivity and specificity.

One member of the Amyloid precursor protein family is APLP2, a type 1 transmembrane glycoprotein [39]. Axon myelination, demyelination, remyelination, and synaptic formation are all modulated by APLP2 [40]. Moreover, APLP2 helps cancer cell survival proliferation, migration and metastasis [41–43]. It has a role in pancreatic cancer [44, 45], colorectal carcinoma [46, 47], Ewing’s sarcoma [48], glioblastoma [49] and renal cell carcinoma [50].

This literature revealed that 100% of non-neoplastic cases showed diffuse moderate to strong nuclear APLP2 positivity with a mean H-score of 209.5. However, all PTC and IEFVPTC showed weak nuclear positivity with a mean H-score of 101.9 and 73.50 respectively. However, these results were contrary to those obtained by Castelblanco et al. who documented APLP2 expression in benign non-neoplastic thyroid tissue in comparison with thyroid carcinoma [15, 51].

Regarding the NIFTP group, 90.3% of our NIFTP cases showed moderate to strong nuclear positivity with 136.1 as a H-score mean. After extensive research, no other literature was found analyzing APLP2 expression in NIFTP.The expression status of the marker didn’t show a significant difference in discrimination between the four studied groups. However, the H-score showed significant differences between the four groups, whereas values of NIFTP were higher than both PTC groups and values of the non-neoplastic group were the highest.

Given using APLP2 as a diagnostic marker discriminating NIFTP from non-neoplastic cases, APLP2 showed high sensitivity to NIFTP at the H-score cut-off point < 250 but at the expense of specificity which is 32.35%. This low specificity can be solved and improved by combining APLP2 with HBME-1 whose specificity for NIFTP against non-neoplastic lesions is 100%. Given differentiating NIFTP from PTC, the best sensitivity for the marker was 78.57% at the H-score cut-off point > 45 with a very low specificity of 11.76%. To discriminate NIFTP from IEFVPTC, the best sensitivity of the marker was 75% and the specificity was 60%. The diagnostic validity of this marker makes it undependable to diagnose NIFTP alone and needs to be combined with other markers with potential diagnostic accuracy like RRM2 and HBME-1.

Combined markers use was tested to increase the diagnostic validity of the markers to NIFTP.In differentiating NIFTP from non-neoplastic lesions, the Combined H score of CD56 and HBME-1 has the highest sensitivity and specificity of 70%, and 96.67% respectively but this sensitivity is lower than using CD56 alone (95%). However, diagnostic accuracy and AUC were improved in this combination compared to CD56 alone. It was also noted that panels with HBME-1 showed the highest specificity. In differentiating NIFTP from PTC, the panel with CD56 had the highest specificity (100%) but the sensitivity is lowered below that of the markers alone. Combined RRM2 and APLP2 had the highest sensitivity (100%) and diagnostic accuracy (93.55%). In differentiating NIFTP from IEFVPTC, the results were highly similar to those of NIFTP against PTC. Panels with CD56 show the highest specificity (90%) and panels of RRM2 show the highest sensitivity. Combining RRM2 with APLP2 improved both sensitivity and specificity above values of APLP2 alone while not showing a significant difference in diagnostic accuracy than using RRM2 alone.

## Conclusion

Although histological evaluation remains the primary method for diagnosing NIFTP, IHC markers may offer additional value in their distinction from other follicular lesions. In differentiating NIFTP from non-neoplastic lesions, CD56 (at an H-score < 225) showed relatively high sensitivity, while HBME-1 appeared more specific. When used in combination, HBME-1 with either CD56 or APLP2 modestly improved specificity and diagnostic accuracy, with the HBME-1 and CD56 pairing showing comparatively better performance than individual markers. For distinguishing NIFTP from malignant lesions, RRM2 demonstrated higher sensitivity, specificity, and diagnostic accuracy within the study cohort. APLP2 showed lower diagnostic performance in differentiating NIFTP from both non-neoplastic and malignant lesions. However, this study has some limitations, including the relatively small sample size and its retrospective design. Further studies with larger cohorts and prospective designs are recommended to validate these findings. Also, molecular studies are recommended to investigate the relation between HBME-1 expression in NIFTP and BRAF mutation. Searching for multiple diagnostic IHC panels for differentiating NIFTP from other follicular thyroid mimickers is required.

## Data Availability

No datasets were generated or analysed during the current study.

## Abbreviations

PTC: Papillary thyroid carcinoma DTC: differentiated thyroid cancer
FVPTC: Follicular variant of PTC NI-EFVPTC: Non-invasive form of encapsulated FVPTC
NIFTP: Non-invasive follicular thyroid neoplasm with papillary-like nuclear features
CK19: Cytokeratin 19 HBME-1:Hector Battifora mesothelial-1 FN1:Fibronectin1
CITED1: Cbp/p300-interacting trans-activator with Glu/Asp-rich carboxy-terminal domain 1 CD56:Cluster of differenitiation 56 GAL3:Galectin3
APLP2: Amyloid precursor-like protein 2 RRM2:Ribonucleotide reductase M2 polypeptide
PRC1: Protein Regulator of cytokinesis 1 WHO: World Health Organization
IEFVPTC: Invasive encapsulated follicular variant papillary thyroid carcinoma
TMA: Tissue Microarray SD: standard deviation IQR: interquartile range
